# Sport and the Covid-19 Pandemic: A Structuralist Analysis of Key Themes in the UK Mass Media

**DOI:** 10.3389/fspor.2020.578472

**Published:** 2020-10-20

**Authors:** Richard Giulianotti, Holly Collison

**Affiliations:** ^1^School of Sport, Exercise and Health Sciences, Loughborough University, Loughborough, United Kingdom; ^2^University of South-Eastern Norway, Bϕ, Norway; ^3^Institute for Sport Business, Loughborough University, London, United Kingdom

**Keywords:** sport, COVID−19, mass media, structuralist analysis, thematic approach

## Abstract

This paper provides a systematic, detailed analysis of UK mass media online reports and narratives on sport and Covid-19 during the main lockdown period over March-May 2020. A “structuralist thematic” approach is utilized to identify and to map systematically the main themes within the mass media. The research is based on reports and narratives on sport-Covid which featured in five leading online UK mass media outlets. The analysis sets out four underpinning statuses or dimensions of sport: the existential, normative, socio-cultural, and political. These dimensions connect directly and, respectively, to four sets of binary opposite media themes on sport during the Covid-19 lockdown: sport as absence/presence, selfish/altruistic, crisis/escape, and threat/solution. Each theme features several types of media report or commentary (which we term “narrative or substantive strands”) on sport-Covid. The paper examines the four binary opposites, and their various types of media report and narrative, in detail. It concludes by discussing the theoretical contributions and substantive findings from the study, and some areas for future research.

## Introduction

The Covid-19 pandemic had an extraordinary impact on world sport, leading to the almost complete shutdown of sports clubs, events, tournaments and businesses, and to the freezing of a global sporting industry valued at over US$500 billion (Business Research Company., 2019). These extreme processes occurred in March-May 2020, during the initial mass spread of the pandemic in Europe and North America, as national governments and the international community implemented, in the words of the International Monetary Fund, a “Great Lockdown,” in the attempt to contain and control the spread of the virus.

In the UK, the pandemic led to the cancellation of many leading sport competitions such as the men's European Championships and various national and regional leagues in football, the Wimbledon tennis tournament, the men's Open golf championship, the Grand National in horse-racing, the London and other city marathons, and the Oxford-Cambridge University boat race. Protracted postponements also arose in men's and women's football, rugby union, and international tours in cricket, golf, tennis, and other sports. At everyday level, UK sport also effectively closed down, with gyms, sport halls, swimming pools, golf courses, and other sport-related venues and facilities being shuttered.

UK sport lockdown measures were put in place as the national government struggled to manage an unfolding disaster in public health. In the main 2-month period (27 March-29 May) of Covid-19's mass spread, during which the UK lockdown also mostly occurred, over 63,000 “excess deaths” were recorded nationally, and the UK was placed second on international mortality measures with respect to the virus^1^.

In regard to academic literature on Covid-19 and sport, it is too early to provide any substantive overview and analysis of published work in this field, particularly research that draws on social scientific perspectives. Published academic work thus far has largely focused on the direct health, social, and organizational impacts of Covid-19 on sport, such as the suspension or cancellation of elite sport events and activities, and debates on their future resumption; how best to provide professional support for elite-level athletes during the lockdown; and, the role of physical exercise and activities in maintaining health and well-being during quarantine (Corsini et al., 2020; Gallego et al., 2020; Jiménez-Pavón et al., 2020; Mohr et al., 2020; Muñoz and Meyer, 2020; Schinke et al., 2020; Toresdahl and Asif, 2020).

One aspect of the sport-Covid relationship that is unlikely to receive much systematic academic investigation involves mass media coverage of the issue. This relative lack of interest may be understandable given the pandemic's pressing impacts in these other fields. However, any continuing lack of research into mass media coverage of the sport-Covid nexus represents a potentially significant academic lacuna for at least four reasons. First, the mass media is a highly important research domain in its own right, as it has become increasingly central to sport, particularly in the intensified globalization and commercialization of elite-level sport, since the early 1990s. Second, during the Covid-19 pandemic and the ensuing lockdowns, the mass media sought to play crucial roles across the public sphere, notably in communicating government instructions and strategies, and scientific advice, to diverse audiences, and also spotlighting the unfolding social disaster that was occurring particularly in hospitals and care homes in the UK context. Third, as we discuss here, the mass media were highly active in seeking to frame and to narrate the sport-Covid interrelationship. This work included disseminating information on changes to the sporting calendar; reporting on the virus's impacts on the sporting world; and, advancing diverse opinions and analyses on how sport was and should be responding to the pandemic. Fourth, at the same time, mass media organizations were required to make large adjustments to their own structures and activities, due to the closure or postponement of so many sporting events, upon which so much of their work, content and revenues are reliant.

This paper therefore seeks to address this research gap by advancing the first systematic, detailed social scientific analysis of mass media reports and narratives on sport and Covid-19. It also contributes more broadly to the growing body of research on the social impacts of Covid-19 on sport. To do so, we utilize a “structuralist thematic” approach in order to identify and to map systematically the main themes of media content vis-à-vis sport and Covid-19 during the main lockdown period in the UK. In turn, the paper develops a theoretical framework and substantive research findings–encapsulated later in Table 1—which may be transferred and applied to undertake further social scientific studies of diverse issues and processes in regard to sport, media or different social crises, in other national or transnational contexts, or in other historical epochs.

**Table 1:** Framework of mass media content on sport and Covid-19.

**Statuses or dimensions of sport**	**Binary opposite themes**	**Narrative or substantive strands**
Existential	Sport as absence	Closure, contraction, contract
	Sport as presence	Reminiscence, anticipation
Normative	Sport as selfish	Exploitative, irresponsible, obscene, evasive
	Sport as altruistic	Welfare, solidarity
Socio-cultural	Sport as crisis	Economy, time, social divisions, infection
	Sport as escape	Nostalgia, compensation, repudiation
Political	Sport as threat	Catalyst, violator
	Sport as solution	Conformist, adapter, mobiliser, and booster

In the following, we begin by outlining the methods used herein for gathering data from online UK mass media and for deploying the structuralist thematic approach for data analysis. We then turn to the substantive part of the paper, which sets out the main themes and types of media report or commentary within each of the four binary oppositions. We conclude by outlining several theoretical contributions and substantive findings from the study, and areas for future research.

## Methods

### Online UK Mass Media

The research for this paper was based on a qualitative investigation of the main themes in the UK mass media with regard to sport and the Covid-19 pandemic. Data was collected mainly during the full UK lockdown period from five leading UK mass media websites: *The Guardian, The Telegraph, Daily Mail, Sun*, and BBC online. These outlets represent a broad sample of UK online mass media: *The Guardian* and *The Telegraph* are leading UK “broadsheet” newspapers with left-of-center and right-of-center respective political standpoints; the *Daily Mail* is a UK high-circulation “middlebrow” tabloid; the *Sun* is the UK's leading mass-market “red-top” tabloid newspaper; and, the BBC is the UK's leading national broadcaster with a strong online presence. One recent survey indicated that these were the five most popular news websites in the UK^2^. While the online media content covered a wide range of sports, most of this material concentrated on men's elite-level football, particularly the English Premier League, which represents the most newsworthy and heavily reported area of UK sport^3^.

The main full UK Covid-19 lockdown period ran from 26 March to 11 May 2020^4^. Lockdown measures initially announced by the UK government on 23 March came into effect 3 days later; imposed for an initial 3 weeks, these measures were extended for a further 3-week period. To reduce the risk of sudden mass unemployment, the UK government introduced a business “furlough” scheme, whereby employees unable to work during the lockdown could be retained by their companies rather than made redundant, with the state paying 80% of wages up to £2,500 per month. On 10 May, the government announced plans to ease the lockdown and reopen parts of society, including encouraging workers in construction and manufacturing to return to work, and lifting restrictions on outdoor exercise^5^.

Elite-level sports in the UK had initially moved at different speeds into initial suspension and then lockdown. In football, the Arsenal-Manchester City Premier League fixture for 10 March was postponed, but the Liverpool-Atletico Madrid Champions League match was played a day later, with 52,000 fans in attendance, including 3,000 from Spain. Two days later, UK professional football was suspended, and other sporting postponements and cancellations followed. However, the 4-day Cheltenham horse-racing festival was staged across 10–13 March and attended by an estimated 150,000 spectators. The IOC's 2020 Olympics boxing qualifying tournament in London, running from 11–24 March, also continued albeit behind closed doors.

### Structuralist Thematic Approach

The data was analyzed using what is termed here as a “structuralist thematic” approach, which combines core structuralist and thematic methodological precepts. In this study, the approach had four main interlocking strands.

First, reports and commentaries on sport and Covid-19 in the relevant UK mass media outlets over the lockdown period were gathered. These reports and commentaries were analyzed inductively with a view toward gradually differentiating and classifying this material, to produce several emerging content themes. The aim was not to generate themes strictly according to basic quantitative criteria, such as the relative frequency of their occurrence in media content. Instead, the concern was to register the broad range and variety of themes in the media reports and commentaries.

Second, we focused on generating themes that are “ideal typical” in the Weberian sense (Weber, 1949). According to Weber (1949, p. 90) definition (emphasis in original):

An ideal type is formed by the one-sided *accentuation* of one or more points of view and by the synthesis of a great many diffuse, discrete, more or less present and occasionally absent *concrete individual* phenomena, which are arranged according to those one-sidedly emphasized viewpoints into a unified *analytical* construct (*Gedankenbild*).

In this context, the ideal types encapsulated specific underlying thematic perspectives (“unified analytical constructs”) on sport vis-à-vis the Covid pandemic. As we explain further below, each ideal-type theme contains several types of story or commentary (“narrative or substantive strands”) which were identified within the online mass media content.

Third, we utilized a structuralist approach to map these ideal-typical themes in a relational way. More specifically, this structuralist approach enables the meanings of these themes to emerge dyadically, through sets of “binary opposite” relations. The use of binary oppositions in social scientific analysis has an extensive multidisciplinary history, having been pioneered by the structural linguistics of de Saussure (1916/2011), and developed subsequently in the structural anthropology of Lévi-Strauss (1963/1967). In sociology, binary or polar opposites are principally associated with structural approaches, such as the structural-functionalism of (Parsons, 1960; see, for example, his “pattern variables”), the systems theory of (Luhmann, 1984/1995; see his use of binary codes), or Bourdieu (1980/1990) structuralist analyses of “logics of practice.” Often, these structuralist dualisms refer to deep cultural, ontological or universal categories. For example, Bourdieu (1980/1990, p. 215) brilliant structuralist analysis of the social ontology of the Kabyle people in northern Algeria is rooted in many fundamental categorical dualisms, such as hot/cold, day/night, dry/wet, outside/inside, and right/left.

Dualisms have been central to the analytical frameworks of many social scientists, perhaps most obviously in fundamental structure/agency debates across the philosophy of social science, as well as in diverse sociological approaches, such as Durkheim (1893/1997) on mechanical/organic solidarity or Tönnies (1887/2001) on social *gemeinschaft/gesellschaft*. More recent examples are provided by the centrality of dualisms in influential theories of globalization, such as Robertson (1992) on the particularism/universalism binary, or Ritzer (2003) on the “glocalization” / “grobalization” couplet.

Structuralist and dualist theoretical frameworks all highlight how the meanings of different concepts (or themes or ideal-types) are inextricably bound to, and reliant upon, their opposites or antonyms. Competitive sport is replete with these binary oppositions—win/lose, team-mate/opponent, home/away, score/miss, fair/foul, and so on—which underpin the logics and meanings of sport participation. Structuralist and system-theory approaches which emphasize these binary codes have been used in diverse ways in the sociology of sport, for example in the study of football fan identities (e.g., Giulianotti, 1991, 2002) or in various applications of Luhmann's theoretical *oeuvre* to sport (Wagner et al., 2010).

Fourth, this structuralist thematic approach, in line with the broader structuralist perspective *per se*, is not focused on diachronic social processes, such as tracing the trajectories or the undulating interplay of these themes over time. The emphasis instead is on identifying and mapping the logics and binary relations of the ideal-type themes that underpinned UK mass media coverage of sport during the Covid-19 lockdown.

## Mass Media, Sport, and COVID-19: Four Binary Opposites

The structuralist thematic analysis of UK online mass media content on sport and Covid-19 yielded a theoretical framework that is encapsulated in Table 1.

To explain this framework, the first column features four deep underlying categorical statuses or dimensions of sport: the existential, normative, socio-cultural, and political. These represent the structural foundations of any domain of sport, and not just the specific area of mass media reports on sport-Covid 19. In this specific area, the four underlying categorical statuses or dimensions connect to four binary oppositions, which are premised upon eight ideal-typical themes. The four binary oppositions center on sport as absent/present, selfish/altruistic, crisis/escape, and threat/solution. Crucially, in each of these binary oppositions, the first theme is a negative one, in which sport is absent, selfish, in crisis, or a threat; the second theme is a positive one, in which sport is present, altruistic, an escape, or a solution. Each ideal-typical theme has several narrative or substantive strands that relate to prominent types of media report, commentary or storyline. It is important also to note the relationships between the categorical statuses of sport and the specific binary opposites: thus, the existential status of sport relates to the absence/presence dualism; the normative status to selfish/altruistic; the socio-cultural status of crisis/escape; and, the political status to threat/solution.

A concise comment is required on the originality of these concepts and overall theoretical architecture. The original aspects here comprise: the overarching theoretical framework that is Table 1; the combination of the four categorical statuses or dimensions within that model; and, the eight ideal-typical themes, four binary opposites, and 24 narrative or substantive strands that have been identified within the mass media reports and contents. Each of the four categorical statuses or dimensions is widely used across social science; however, our identification of each dimension with respect to the mass media reports and contents is also original.

The eight themes, four dimensions, and 24 strands are intended to encapsulate the breadth of all sport-Covid mass media narratives that were identified and subjected to inductive thematic analysis. However, two brief caveats are required on the extent to which this represents the “full story” in terms of mass media content and the development of the theoretical framework. First, during the initial gathering of data, some possible media stories were discounted on the grounds that their discussions of sport and/or Covid-19 were relatively minor. Second, we recognize that future analyses of media content by other researchers may lead to the generation of further themes, dimensions, binary opposites, and strands. Such critical developments of analytical frameworks are standard processes in theory-building.

We turn now to set out in some detail the four binary opposites, and their eight ideal-typical themes, before providing some concluding comments.

### Sport as Absence/Sport as Presence

The first binary opposition is a fundamental one—centered on the existence of sport—and appears here as the “base” dualism upon which the other couplets are founded.

#### Sport as Absence: Closure, Contraction, Contract

Inevitably, the most conspicuous media theme related to the absence of sport, which harbored several strands relating to issues of closure, contraction, and contract.

A very large volume of coverage was given to the *closure* of elite sport leagues and competitions, both in the UK and internationally. By the start of the UK lockdown, online media were providing checklist reports on how different sports—such as football, rugby union, rugby league, tennis, golf, netball, and marathon races—had been suspended or shut down^6^. Some reports highlighted the historical exceptionality of these measures, with sports events such as Wimbledon being called off for the first time in peacetime^7^. Additional focus was directed to the shuttering of sport clubs, golf courses, gyms and betting shops at everyday level^8^.

Second, the actual volume of sport content in online media, and in the wider sport-media system, *contracted* very sharply as event cancellations and postponements took effect. While there was a lack of daily sport events on which to report, general sport analysis and opinion articles also declined, with little scope for standard background news stories, such as on football player transfers and managerial sackings. Sport analysis and opinion columns, written by leading sport-writers or top former athletes, were also absent or reduced in scale.

Third, online media reports also focused on the absence of sport content in the wider media, particularly television networks, and on the subsequent *contractual* implications for consumers as well as sport clubs and federations. Pay-TV networks such as Sky and BT Sport, whose whole business model was premised on entire television stations devoted to live sport, lost almost all of this crucial content during the lockdown, and sought to plug this gap temporarily with documentaries and old highlights. The online news narrative here centered on protecting the contractual rights of pay-TV consumers: several stories focused critically on how Sky and BT Sport had refused to offer paying customers refunds for subscriptions which had been sold on the promised provision of live sport content^9^. Subsequent reports pointed to the possibility of consumers suspending their subscriptions until live sport action, particularly in English Premier League football, was able to return^10^.

#### Sport as Presence: Reminiscence and Anticipation

At the other end of this continuum, the theme of sport's *presence* was manifested in two main ways. This occurred not with respect to the present, but instead, somewhat paradoxically, with regard to the past and looking back (reminiscence), and to the future and looking forward (anticipation).

First, sport memories and nostalgia were prominent, as sport media activated and recycled old sporting material to fill emptied television schedules and webpages. The BBC were candid on their approach: as its Director of Sport, Barbara Slater, stated, “In these unprecedented and difficult times we are delighted to bring some of the most incredible sporting events from years gone by to our audiences over the next few months” (*The Guardian*, 2 April 2020)^11^. In this vein, online media turned to *reminiscence* articles and “as live” coverage of sport events. For example, *The Guardian* initiated a regular column, “My Favorite Game,” in which contributors reminisced on their best sporting experiences; the newspaper also ran “minute-by-minute” live-style text reports on classic sport events, such as the 1978 Scotland-Netherlands World Cup football fixture, the 1986 and 1996 Masters golf tournaments, and the 1977 Grand National in horse-racing^12^. BBC Sport's website adopted a similar approach by streaming films of old sport events. The deaths of former footballers Peter Bonetti and Norman Hunter in this period inspired more vivid, poignant content, with reminiscences on their epic matches, notably the notoriously violent Chelsea-Leeds United FA Cup final replay in 1970^13^.

Second, there was substantial *anticipation* of the “grand return” of sport, particularly English Premier League football. This wide-ranging narrative took a variety of angles, including: speculation on the projected dates of sporting return, and how player or athlete training and subsequent events would be staged; concerns for the safety of participants whether in training or competitive activities; disputes between various competitors, clubs and sport governing bodies over how sport competitions might be altered to enable their completion, for example with football matches contested in neutral stadiums; reports on how post-Covid sports were being staged in other locations, such as Bundesliga football in Germany, or horse-racing in Hong Kong, and what lessons might be drawn for the UK; and, the economic imperatives for elite sport leagues to return in order to protect their income from television^14^. Overall, this diverse anticipation of sporting return provided a long-running, shifting narrative in media coverage of sport throughout the lockdown.

### Sport as Selfish/Sport as Altruistic

The second couplet—centered on the normative aspects of sport—relates to the binary themes of sport as selfish and as altruistic.

#### Sport as Selfish: Exploitative, Irresponsible, Obscene, Evasive

The “sport as selfish” theme was underpinned largely by wider public and political arguments on sport's extensive commercialization in recent decades, with the perceived resulting tendency to prioritize commercial interests over social, welfare, and ethical issues. Four main critical strands were prominent in online media reports and analyses.

First, several sport clubs were accused of *exploitative* and greedy industrial practices as the lockdown took effect. English Premier League clubs were the most common target, reflecting a regular media unease over the perceived excessive commercialism among its owners and financial leaders. For example, Mike Ashley—owner of Newcastle United and the Sports Direct merchandise company, whose business practices often attracted political condemnation and public protests—was criticized for initially seeking to keep his retail stores open during the lockdown, then allegedly pressing staff into working despite their being classified as “furloughed” (non-working)^15^. Other football clubs such as Tottenham Hotspur and, in particular, Liverpool, were heavily criticized for placing staff on furlough, to avoid their billionaire owners incurring salary costs^16^.

Second, some sport governing bodies were criticized for their attempts to continue staging events and tournaments despite the pandemic. The IOC were criticized as “*irresponsible”* by some boxing federations after the Olympic boxing tournament was continued as the pandemic took hold, with the result that some participants contracted Covid-19^17^. The IOC leadership attracted further protests from many athletes and some sport federations for delaying the seemingly unavoidable decision, to postpone the Tokyo 2020 Olympics^18^. Cheltenham festival organizers also faced criticism for staging their event as the pandemic took hold^19^.

Third, the rationale for the proposed return of English Premier League club football came under scrutiny. Criticisms here centered on the essentially financial motives for the return—the risk of losing £750 million in contracted television money—rather than prioritizing more important factors such as the safety of players and fans, the integrity of sporting competition, or broader support for and protection of the NHS^20^. The initial English football plan, to recommence fixtures on 30 April during lockdown, was castigated as “*obscene”* by Oliver Holt, a prominent UK sportswriter, in the context of a national health emergency:

All that April 30 date did was reinforce the worst opinions of the Premier League: that it is a league in denial; a league so bloated with cash that it is out of touch with reality and that it deludes itself that, while hospitals and their staff fight against the pandemic and the body count rises, the public harbor a desire to return to its stadiums^21^.

Similar accusations of greedy or obscene commercial priorities were directed toward the controllers of non-league football in England, in allowing games to take place, and Formula 1 in seeking to stage the Australian Grand Prix as the global pandemic intensified^22^.

Fourth, a related aspect here involved demands for highly paid sport stars not to *evade* their responsibilities, and to contribute to the national effort by accepting pay cuts. The UK's Minister for Health, Matt Hancock, and other commentators argued that highly rewarded football players should follow this course of action^23^.

#### Sport as Altruistic: Welfare and Solidarity

At the other side of this continuum, the theme of sport's altruism and social responsibility was advanced in two main ways. First, as sport events were called off, and as the lockdown took effect, UK sport officials were reported as stating that their priorities lay with the health and *welfare* of athletes and the wider public rather than with any other concerns. In men's rugby union, clubs emphasized their commitment to give their players the best possible support and resources during lockdown^24^. The Women's Netball Players' Association launched a mental health campaign early in the pandemic, to support athletes who had been forced to suspend their work and were also facing the threat of salary freezes^25^.

Second, the *solidarity* of sport stars in fund-raising, donations, and social support was given significant discussion. In men's football, this included fund-raising initiatives such as the “#PlayersTogether” campaign, in support of front-line NHS workers, which also represented a pointed response to government ministers and media commentators that these stars were not “doing their bit”^26^. The voluntary work of athletes was also highlighted, such as the Welsh rugby union international Jamie Roberts, and Olympic medallist Gail Emms, in working to support the National Health Service (NHS)^27^.

### Sport as Crisis, Sport as Escape

The third couplet—centered on the socio-cultural aspects of sport—concerned sport's double-edged relationship to Covid-19: as a site of crisis, ensnared by the pandemic; and, as a site of escape, where the pandemic could be side-stepped.

#### Sport as Crisis: Economy, Time, Social Divisions, Infection

In regard to crisis, four intersecting strands were evident in online media reports on how sport was a site of emergency in the midst of the pandemic. First, the devastating *economic* impact of the pandemic on sport as well as in other areas of the global economy was accorded substantial media coverage. Again, elite men's football attracted substantial focus, with the Premier League under pressure to complete the season of 92 remaining fixtures, or face the loss of television revenue estimated in some reports as totalling up to £1.5 billion^28^. Yet the most significant crises were faced by much smaller and more modest sport federations. Several leading sport governing bodies were reported as facing “meltdown,” with a combined “£740 million financial black hole”; cricket alone faced losses of around £380 million (*The Telegraph*, 5 May 2020)^29^. A particular concern centered on women's sports, which had made substantial progress in recent years, but faced being to the fore in any financial cutbacks^30^.

Second, a continuing story centered on the unfolding devastation wrought by the pandemic on sporting *time*; that is, on the sporting calendar. Regular media updates followed on how a steady stream of major events—such as the 2020 Olympics, the 2020 men's football European championships, the Six Nations rugby championship, and further international tournaments in cricket, golf, rugby league, tennis, athletics, baseball, Formula 1 motor racing, and other sports—had been canceled or given long-term postponement^31^.

Third, the pandemic exposed and exacerbated *social divisions* within sport and wider society. There was substantial political, scientific and media focus on Covid-19's threat to specific population groups, particularly with respect to higher mortality levels according to age (older people), disability, gender (male), ethnicity (BAME groups), and those with underlying health conditions; and, on how disadvantaged communities were struggling to manage during the lockdown^32^. In sport, there was significant focus on Covid-19's threat to BAME athletes, who constituted a relatively high proportion of elite-level performers, particularly in football, and the greater risks faced by these stars during any proposed return to training and competition^33^. Another focus was on how, at everyday levels, women had been adversely affected by the lockdown, for example through drops in their levels of physical activity and exercise^34^.

Finally, media stories also focused on those in the sporting world who had endured viral *infection*. The most extreme cases involved victims who had died from the virus, such as the former Leeds United and England footballer, Norman Hunter, the Italian Olympic runner Donato Sabia, and the former Real Madrid President, Lorenzo Sanz^35^. A more common set of stories centered on contemporary athletes and coaches, particularly within team contexts, who had been diagnosed positive and were required to manage the virus^36^.

#### Sport as Escape: Nostalgia, Compensation, Repudiation

At the other side of this continuum, some focus was given over to sport as escape, in terms of evading the impacts of Covid-19. Three key aspects of this theme centered on nostalgia, compensation and repudiation.

First, as noted earlier, sporting reminiscences within the media offered a collective psycho-cultural escape from the lockdown, enabling viewers and readers to revel in the *nostalgia* of classic moments from the pre-Covid sporting era.

Second, some media attention turned to *compensatory* forms of sport involvement. Particular focus was on how e-sports were reportedly “booming” in popularity, as sport audiences sought competitive if simulated sport participation during the lockdown^37^. Virtual and actual sports were bridged by stories that some sport stars during the lockdown were engaged in e-sport competitions, such as various tennis stars competing in a virtual Madrid Open, or Joe Frazier vs. Lennox Lewis in online boxing^38^. Elite-level darts was most successful in combining actual and virtual formats, as competitors played in their own homes, while being connected over livestream broadband video links. The initial competition was complicated by a weak wifi connection at the home of former world champion, Gary Anderson, which disrupted his participation^39^.

Third, reports focused on how some nations had *repudiated* global public health guidance, ignoring the pandemic, and continuing to stage sporting competitions as well as other substantial public gatherings. The most extreme example was provided by Belarus, where President Alexander Lukashenko refused to cancel any events, and highlighted his commitment to these policies by participating in an ice-hockey match^40^. As sport events across the world went into lockdown, the Belarus national men's football league also continued to stage fixtures^41^.

### Sport as Threat, Sport as Solution

The fourth dualism—centered on the political aspects of sport—related to online media coverage of sport as a threat and as a potential solution to the pandemic.

#### Sport as Threat: Catalyst and Violator

There were two main strands to the theme of sport as a potential threat to health and well-being. First, the role of sport as a *catalyst* for spreading the virus was given significant focus. As noted earlier, at elite level, attention turned to two specific events: the Cheltenham horse-racing festival (10–13 March), attended by around 150,000 people; and, the Liverpool-Atletico Madrid Champions League fixture (11 March). Some reports pointed toward spikes in local mortality rates 20–35 days after these events, leading to calls for expert inquiries into their actual health impacts^42^.

Sport-related holidays and other social activities at everyday level were spotlighted as potential catalysts for spreading the disease. Some of the earliest potential Covid-19 infections of UK citizens—including the possible UK “patient zero” —were traced to the Austrian skiing resort of Ischgl, where après-ski parties were reported to have afforded an ideal environment for viral diffusion^43^.

A second aspect to the sport-as-threat theme related to how sport might *violate* government instructions on social behavior during the Covid-19 lockdown, with the potential for the infection spreading across the wider population. This media storyline tapped into a classic media narrative, centered on exposés of the misbehavior of sport stars. Thus, for example, football players were criticized for “flouting” social distancing rules by training together, and rugby players for meeting socially^44^. More salacious stories followed, for example on one men's football player who reportedly hosted a sex party a day after his social media post had urged people to practice social distancing^45^.

#### Sport as Solution: Conformist, Adapter, Mobiliser, Booster

At the other side, the theme of sport as a potential social solution during the pandemic carried four main strands. First, sport's *conformity* was highlighted, in following or offering support for government instructions and scientific guidance during the pandemic. Under lockdown conditions, the public were advised to take sporting exercise in open spaces, for example cross-country running and mountain-biking^46^. As the prospective return of elite-level sport was discussed, sport federations, clubs, and athletes were advised on how training could begin, while maintaining social distancing along with the deep-cleaning of sport facilities and equipment^47^.

Second, the *adaptations* of elite-level sport, in protecting against the virus during returns to action, were given substantial coverage. These measures included the extensive testing of potential participants for Covid-19, and the staging of events “behind closed doors” (sometimes known as “ghost games”) in sports such as horse-racing, football, golf, and cricket^48^. In elite men's football, substantial interest centered on so-called “ghost games” in which stadiums and venues were emptied of all spectators. Meanwhile, elite sport leagues and clubs put in place safeguarding and support systems in order to protect players and staff, and to advance the prospect for a smooth return to action. Athletes and other staff who contracted Covid-19 were self-isolating at home, usually with mild symptoms^49^.

Third, at everyday level, the importance of sport and physical activity for *mobilizing* the nation, to keep the public fit during and after lockdown, was given prominence. There was substantial media guidance on effective home fitness regimes, while linking to workouts available on social media, such as those offered by celebrity trainers such as Joe Wicks^50^. The UK government was also pressed to permit gyms and other fitness sites to reopen, in part to tackle potential rises in obesity which in turn could increase the risk of Covid-19 infections becoming more serious^51^.

Finally, the role of elite-level sport, and in particular English men's football, as a *booster* for the national mood was spotlighted. At the pandemic's outset, some sport commentators argued that the future return of football “could bring some joy back to the nation”^52^. The England cricket captain—the Irishman, Eoin Morgan—commented that, “Playing cricket again, even behind closed doors, would help boost the nation's morale”^53^. Later, as the UK government assessed easing the lockdown, foreign secretary Dominic Raab commented that the return of sport would “lift the spirits of the nation”^54^.

## Discussion and Conclusion

In this paper, we have utilized a “structuralist thematic” approach to organize and to analyse UK online mass media coverage of sport during the Covid-19 lockdown. The analytical framework and substantive findings are summarized in Table 1. The analysis is anchored in four underpinning statuses or dimensions: the existential, normative, socio-cultural, and political statuses of sport. These dimensions connect directly to four sets of binary opposites that feature a total of eight themes: sport as absence/presence, selfish/altruistic, crisis/escape, and threat/solution. Each theme features several narrative or substantive strands that are associated with particular types of sport-Covid media report or commentary.

To conclude, we wish to make three sets of observations which relate, respectively, to the substantive themes within the model, as set out in Table 1; to the use of the model in future research; and, briefly, to broader substantive and theoretical insights on sport and the mass media which follow from the paper.

To begin, we consider the substantive themes within the model; three main points might be made here. First, it is evident that the negative themes within the model—which present sport as absent, selfish, in crisis, or a threat—tend to relate to economic and health questions, and to portray sport as having an excessive self-importance. Thus, for example, there is significant focus on Covid-19's crippling impacts on sport's finances, and on the excessive self-interest of some leading sport figures, clubs, and governing bodies in thinking of their commercial concerns when responding to the crisis.

Second, the positive themes—that present sport as present, altruistic, an escape, and solution—tend to be associated with its psychological and social benefits. These positive themes also point to key sport stakeholders being relatively humble, or appreciative of their secondary importance in the midst of crisis. Thus, for example, online media reports included focus on how sporting reminiscences, esports, and the anticipation of sport's return, were evident during the lockdown, potentially offering some escapist, compensatory, or morale-boosting benefits for individuals and social groups during the crisis. Media reports also spotlighted sport's contribution to the “greater good” in terms of fund-raising, voluntary work, and other forms of social support.

Third, there is a further, underlying difference between the positive and negative themes within the binary oppositions, and their respective narrative or substantive strands in mass media coverage of sport/Covid. Negative substantive strands tend to highlight the mundane impacts and material realities of the pandemic in relation to sport: the closure or contraction of sport, its economic and temporal shocks, its intensification of social divisions, and its role in catalyzing viral transmission, for example. Positive substantive strands have a markedly different tone and emphasis, in highlighting critical responses and alternative social arrangements, particularly in the imaginings of such arrangements within media narratives on sport and Covid-19. Thus, for example, there are reminiscence, nostalgia, and anticipation in imagining sport, in past and future; and, sport's role in shaping new societal ways forward, in terms of welfare, solidarity, mobilization, and boosting public morale. Future research into mass media discourses on crisis situations, whether in sport or in other social fields, might investigate further these differences between negative and positive strands in media content.

We turn now to address more fully the second concluding issue, on how future research on the model may be developed. Four main points arise here. First, the overall model provides the basis for further systematic research into mass media narratives with respect to sport or other social fields, such as in the arts, consumerism, industry, travel and tourism, and the academic world. This research may focus on context of Covid-19, or other transnational lockdowns such as during the First and Second World Wars, or other global shocks such as the 2008 financial crisis. It may also be internationally comparative, for example by exploring how mass media in other parts of the world have reported on sport during the Covid-19 lockdown.

Second, future sociological research on any sporting domain may be underpinned analytically by the underlying statuses of sport—the existential, the normative, the socio-cultural, and the political—which have been set out here. For example, the various research fields of sport and the mass media, spectator cultures and identities, athlete migration, the governance and management of sport, the sport for development sector, and sport, physical activity, and health, are all domains in which the existential, normative, socio-cultural, and political dimensions of sport are in place. Accordingly, these latter four statuses may provide the analytical starting-points for research in these fields.

Third, future research may also be guided by the eight themes within the binary oppositions, and their respective substantive strands. Again, there is particular scope here for comparative research, for example in examining how the mass media from outside the UK have reported on sport-Covid, and what similar or differing types of binary opposition and substantive strand may be identified within this non-UK media content.

Fourth, in theoretical terms, there is certainly scope for the model to be further developed in ways that have been precluded here by the pressures of brevity. From a structuralist perspective, one future focus may relate to the binary oppositions, and specifically to the intermediary points between these dualisms. This focus would involve research into the thresholds, liminal spaces, and transitional zones of each dualism; and, into the special “rites of passage” that would mark any movements between one polarity and the other. Hence, to pick one example, in relation to the sport as selfish/altruistic couplet, research might examine media reports and commentaries that moved between these two themes, and explore what may serve to catalyze any shifts in media emphasis from “sport as selfish” to “sport as altruistic” and vice-versa over time. A further diachronic aspect may be added by examining the substantive strands of media reports, to explore how, for example, media discourses move between positive and negative substantive strands; what patterns may be identified in these movements between particular strands; and, what factors tend to influence these shifts in emphasis.

Finally, we turn to explore some broader substantive and theoretical issues on sport and the media. While these broader issues are generally beyond the scope of the paper, three emergent points may be forwarded. First, the research here does highlight how, amidst the huge national shock of the Covid-19 pandemic lockdown, much of the mass media reverted to long-established, tried-and-tested storylines, narratives, and discourses. These included the implicit claims of media outlets to be acting in the public interest by promoting collective solidarity, public health and welfare, and issues of national concern; and, media stories and opinion pieces which featured exposés and trenchant criticisms of sport celebrities and authorities.

Second, there are also clear indications of how the mass media outlets examined here—the UK's leading newspapers and broadcasters—position themselves pragmatically within the wider multi-platform media and communication environment, alongside for example online outlets, social media, and gaming and e-sports. This pragmatism is demonstrated most obviously by the very extensive online news presence of the five media organizations in this study. It is also highlighted by how these media behemoths co-exist with and draw upon these other media platforms, for example through engaging with social media content or reporting on e-sport activities.

Third, the mass media narratives point to the potential utility of postmodern social theories for examining key aspects of the sport-Covid and media interface. Particularly relevant aspects of postmodern social theory here relate to the focus on the virtual world and the deconstruction of social categories^55^. In the sport-Covid context, these postmodern aspects are evidenced by, for example, media emphasis on televised, online, and other “virtual” forms of sport, rather than “actual” sport attendance; and, how athletes turned to e-sports due to the cancellation of “real” sport events. These insights on postmodern trends in sport media fit with other studies which highlight how fans move into other categorical roles—as producers, participants, and broadcasters—within contemporary sport (Andrews and Ritzer, 2018; Majumdar and Naha, 2020; Sturm, 2020). They also remind researchers of the creative possibilities and enduring value of particular social theories—such as postmodernism or structuralism—for examining contemporary transformations in social fields such as sport and the media.

## Data Availability Statement

The original contributions presented in the study are included in the article/supplementary material, further inquiries can be directed to the corresponding author/s.

## Author Contributions

All authors listed have made a substantial, direct and intellectual contribution to the work, and approved it for publication.

## Conflict of Interest

The authors declare that the research was conducted in the absence of any commercial or financial relationships that could be construed as a potential conflict of interest.

## Footnotes

^1^See https://www.theguardian.com/society/2020/jun/09/excess-deaths-in-uk-under-coronavirus-lockdown-pass-63,000; https://www.ft.com/content/6b4c784e-c259-4ca4-9a82-648ffde71bf0

^2^See https://www.statista.com/statistics/376297/news-sites-ranked-by-unique-audience-in-the-uk/ The order here was BBC, *Sun, Daily Mail, The Guardian, The Telegraph*.

^3^The accessibility of all online reports referred to in this paper was checked and confirmed on 26 June 2020.

^4^Lockdown measures during this period required people in the UK to stay at home; avoid all “unnecessary journeys or social contact” beyond their households; take only one form of outdoor exercise each day; leave home only for “essential shopping or medical needs”; avoid traveling to work unless “absolutely necessary”; and, when interacting with people from outside of their household, maintain social distancing of at least two meters.

^5^https://www.theguardian.com/world/2020/may/10/uk-coronavirus-lockdown-what-has-boris-johnson-announced

^6^See for example https://www.telegraph.co.uk/sport/0/coronavirus-cancelled-premier-league-six-nations-london-marathon-2020-postponed/; https://www.bbc.co.uk/sport/51605235

^7^https://www.dailymail.co.uk/sport/sportsnews/article-8175005/Coronavirus-UK-Wimbledon-tennis-CANCELED-time-World-War-II.htm

^8^See for example https://www.thesun.co.uk/news/uknews/11261387/are-gyms-closed-due-to-coronavirus-puregym-fitness-first-and-virgin-active-updates/; https://www.bbc.co.uk/sport/golf/52018267

^9^https://www.thesun.co.uk/sport/football/11170011/sky-sports-refund-coronavirus-bt-sport/; https://www.thesun.co.uk/sport/football/11197598/sky-sports-customers-fuming-subscriptions-coronavirus/; https://www.dailymail.co.uk/sport/sportsnews/article-8112803/Sky-Sports-BT-refunds-coronavirus.html

^10^See https://www.dailymail.co.uk/sport/othersports/article-8133947/How-pause-Sky-Sports-subscription-FREE-amid-coronavirus-outbreak.html

^11^See https://www.theguardian.com/sport/2020/apr/02/bbc-summer-sport-nostalgia-coronavirus-lockdown

^12^See https://www.theguardian.com/sport/sport+tone/minutebyminute

^13^See https://www.theguardian.com/football/live/2020/mar/21/chelsea-v-leeds-united-1970-fa-cup-final-replay-live?page=with:block-5e74a87f8f088d7575597188; https://www.bbc.co.uk/sport/football/52416192

^14^https://www.bbc.co.uk/sport/football/52326617; https://www.dailymail.co.uk/sport/sportsnews/article-8287775/Bundesliga-return-15-German-government-gives-green-light.html; https://www.bbc.co.uk/sport/horse-racing/52698288; https://www.telegraph.co.uk/sport/2020/04/10/scientists-warn-crowds-sporting-events-may-not-return-august/; https://www.telegraph.co.uk/football/2020/04/29/police-will-call-football-season-fans-try-gather-watch-games/; https://www.dailymail.co.uk/sport/football/article-8260747/Here-dates-fans-diary-officials-plot-footballs-return-COVID-19-lockdown.html; https://www.thesun.co.uk/sport/football/11484857/arsenal-return-training-own-pitch-coronavirus/; https://www.theguardian.com/football/2020/may/08/neutral-venue-plan-has-no-rationale-says-former-football-police-commander-premier-league-fans; https://www.dailymail.co.uk/sport/cricket/article-8270277/Moeen-Ali-believes-coronavirus-crisis-drastically-better-cricket-return.html

^15^https://www.thetimes.co.uk/article/ashley-eats-humble-pie-after-row-with-staff-public-and-government-p5927k25s; https://www.theguardian.com/business/2020/may/03/sports-direct-managers-accuse-firm-of-making-them-work-on-furlough

^16^https://www.dailymail.co.uk/sport/football/article-8191989/Coronavirus-Wealthy-owners-Liverpool-Spurs-Newcastle-excelled-selfish-stakes.html; https://www.thesun.co.uk/sport/football/11327925/liverpool-place-staff-furlough-coronavirus/

^17^https://www.dailymail.co.uk/sport/olympics/article-8153837/Turkish-Boxing-Federation-slams-irresponsible-IOC-two-boxers-contract-coronavirus.html

^18^https://www.theguardian.com/sport/2020/mar/25/tokyo-2020-olynpics-thomas-bach-donald-trump-coronavirus-delay

^19^https://www.dailymail.co.uk/sport/racing/article-8234987/Racing-chief-hints-early-return-despite-sport-facing-criticism-staging-Cheltenham-Festival.html

^20^https://www.thesun.co.uk/sport/football/11291528/prem-clubs-season-restart-may-coronavirus/; https://www.bbc.co.uk/sport/football/52630497; https://www.theguardian.com/sport/2020/mar/14/sport-coronavirus-empty-schedules-football; https://www.theguardian.com/football/blog/2020/apr/25/finishing-premier-league-season-pointless-football

^21^See https://www.dailymail.co.uk/sport/football/article-8163369/OLIVER-HOLT-Premier-League-NHS-100m-use-pay-agents.html

^22^https://www.theguardian.com/sport/2020/mar/14/sport-coronavirus-empty-schedules-football; https://www.thesun.co.uk/sport/motorsport/11155713/lewis-hamilton-coronavirus-f1-australia/

^23^See https://www.bbc.co.uk/sport/football/52142267; https://www.telegraph.co.uk/football/2020/03/27/premier-league-players-may-forced-take-50-per-cent-pay-cuts/

^24^See https://www.telegraph.co.uk/rugby-union/2020/03/27/harlequins-helping-players-physical-social-mental-health-lockdown/;

^25^https://www.telegraph.co.uk/netball/2020/03/25/netball-players-association-launches-coronavirus-mental-health/

^26^See https://www.telegraph.co.uk/football/2020/03/25/premier-league-stars-back-campaign-raise-100000-local-communities/; https://www.bbc.co.uk/sport/england/52037307; https://www.dailymail.co.uk/sport/sportsnews/article-8188885/Coronavirus-Wayne-Rooney-slams-Matt-Hancock-claims-players-turned-scapegoats.html https://www.dailymail.co.uk/sport/football/article-8234539/Premier-League-playerstogether-initiative-raises-4MILLION-NHS-launch.html; https://www.thesun.co.uk/sport/football/11358506/premier-league-captains-donation-nhs-coronavirus/; https://www.dailymail.co.uk/sport/sportsnews/article-8206389/Stars-stepped-help-coronavirus-pandemic.html

^27^https://www.bbc.co.uk/sport/rugby-union/52205287; https://www.thesun.co.uk/sport/11670791/gail-emms-coronavirus-olympic-volunteer/

^28^https://www.thesun.co.uk/sport/football/11591621/premier-league-clubs-tv-money-broadcastersgle-coronavirus/

^29^https://www.telegraph.co.uk/sport/2020/05/05/sports-darkest-hour-740m-black-hole-governing-bodies-budgets/

^30^https://www.dailymail.co.uk/sport/sportsnews/article-8341739/Womens-sport-left-lockdown-Fears-cuts-teams-fold-livelihoods-lost.html

^31^https://www.telegraph.co.uk/sport/0/coronavirus-cancelled-premier-league-six-nations-london-marathon-2020-postponed/; https://www.bbc.co.uk/sport/51605235

^32^https://www.theguardian.com/world/2020/apr/22/why-are-people-from-bame-groups-dying-disproportionately-of-covid-19; https://www.telegraph.co.uk/news/2020/04/29/over-50s-should-kept-coronavirus-lockdown-measures-longer-say/; https://www.dailymail.co.uk/health/article-8303307/Vulnerable-Britons-stripped-social-care-Covid-19-lockdown.html

^33^https://www.dailymail.co.uk/sport/sportsnews/article-8336853/PFA-calls-research-coronavirus-affects-BAME-stars.html; https://www.theguardian.com/sport/2020/may/08/shock-fear-and-unease-loom-over-bame-athletes-before-return-to-action-covid-19

^34^https://www.telegraph.co.uk/sport/2020/04/30/women-disproportionately-impacted-fall-activity-levels-lockdown/

^35^https://www.theguardian.com/sport/2020/apr/08/italian-olympic-800m-finalist-donato-sabia-dies-of-coronavirus-aged-56; https://www.telegraph.co.uk/football/2020/03/21/former-real-madrid-president-lorenzo-sanz-dies-contracting-coronavirus/

^36^https://www.bbc.co.uk/sport/football/52605961; https://www.bbc.co.uk/sport/rugby-union/52325189

^37^https://www.telegraph.co.uk/technology/2020/04/08/boom-esports-fans-try-fill-void-left-decimated-sporting-schedule/; https://www.theguardian.com/sport/2020/apr/11/esports-ride-crest-of-a-wave-as-figures-rocket-during-covid-19-crisis.

^38^https://www.dailymail.co.uk/sport/sportsnews/article-8203991/Andy-Murray-virtual-Madrid-Open-month-bid-raise-money-coronavirus-pandemic; html; https://www.telegraph.co.uk/formula-1/2020/03/20/formula-1-replace-postponed-races-virtual-grand-prix-series/; https://www.bbc.co.uk/news/technology-52049893

^39^https://www.bbc.co.uk/sport/darts/52654017

^40^https://www.bbc.co.uk/sport/av/52094552

^41^https://www.telegraph.co.uk/news/2020/04/22/belarus-denies-danger-coronavirus-outbreak-football-matches/

^42^https://www.telegraph.co.uk/global-health/science-and-disease/revealed-three-uk-sporting-events-may-have-led-coronavirus-death/; https://www.theguardian.com/sport/2020/apr/21/experts-inquiry-cheltenham-festival-coronavirus-deaths

^43^https://www.telegraph.co.uk/global-health/science-and-disease/uk-patient-zero-east-sussex-family-may-have-infected-coronavirus/; https://www.bbc.co.uk/news/world-europe-52384572; https://www.dailymail.co.uk/news/article-8154067/UKs-patient-zero-Sussex-worker-50-caught-corornavirus-Austrian-ski-resort-party-bar.html

^44^https://www.telegraph.co.uk/football/2020/04/21/spurs-forced-remind-players-social-distancing-guidelines-serge/; https://www.theguardian.com/sport/2020/may/07/saracens-apologize-after-billy-vunipola-and-teammates-break-lockdown-rules-rugby-union

^45^https://www.thesun.co.uk/sport/11329263/kyle-walker-sex-party-coronavirus-lockdown-hookers-man-city/

^46^https://www.bbc.co.uk/news/uk-england-51943531

^47^https://www.bbc.co.uk/sport/52652893

^48^https://www.dailymail.co.uk/sport/football/article-8287831/Premier-League-horse-racing-return-regular-testing-conducted-says-Matt-Hancock.html; https://www.telegraph.co.uk/football/2020/05/11/sporting-events-behind-closed-doors-return-june-boost-project/; https://www.telegraph.co.uk/sport/2020/05/18/ghost-stadiums-prove-professional-sport-needs-audience/; https://www.dailymail.co.uk/sport/sportsnews/article-8260281/Coronavirus-UK-Premier-League-estimates-300-people-needed-match.html

^49^https://www.bbc.co.uk/sport/cricket/51915496; https://www.thesun.co.uk/sport/football/11182427/footballers-self-isolation-gyms-keeping-fit/; https://www.theguardian.com/football/2020/mar/28/eight-west-ham-players-in-self-isolation-with-mild-coronavirus-symptoms

^50^https://www.dailymail.co.uk/femail/article-8167269/Revealed-Effective-home-workout-complete-lock-down.html

^51^https://www.telegraph.co.uk/news/2020/05/08/pm-urged-allow-gym-reopening-exercise-wonder-drug-coronavirus/

^52^https://www.dailymail.co.uk/sport/football/article-8132771/Coronavirus-UK-Gary-Neville-says-festival-football-bring-hope-joy-outbreak.html

^53^https://www.thesun.co.uk/sport/11305330/eoin-morgan-england-cricket-coronavirus-hope/

^54^https://www.bbc.co.uk/sport/52552667;

^55^For a discussion of postmodern social theories in relation to sport, see Giulianotti (2015).
